# Long-term responses of riparian plants’ composition to water level fluctuation in China's Three Gorges Reservoir

**DOI:** 10.1371/journal.pone.0207689

**Published:** 2018-11-28

**Authors:** Zunji Jian, Fanqiang Ma, Quanshui Guo, Aili Qin, Wenfa Xiao

**Affiliations:** Key Laboratory of Forest Ecology and Environment of State Forestry Administration, Research Institute of Forest Ecology, Environment and Protection, Chinese Academy of Forestry, Haidian, Beijing, PR China; Shandong University, CHINA

## Abstract

The water level fluctuation zone (WLFZ) has experienced a novel hydrological regime due to the anti-seasonal operation of China’s Three Gorges Reservoir. Overall, hydrological change can significantly influence the riparian environment and shift the riparian vegetation. Although numerous studies have investigated the short-term responses of riparian plants to water level fluctuation in this zone, few have addressed long-term effects. In this study, four permanent plots in the WLFZ of the canyon landform area were chosen to evaluate the long-term responses of riparian plants to water level fluctuation from 2008 to 2015 and to screen candidate plants for ecological restoration. We recorded 146 species in 2008, 110 species in 2009, 68 species in 2012 and 69 species in 2015, indicating a conspicuous loss in riparian plants. Most of the remnant plants were annual and perennial herbs. Of the native species present in 2008, 82, 22 and 8 had disappeared in 2009, 2012 and 2015, respectively. Simultaneously, 45, 15 and 11 non-native species were first found, respectively. Additionally, over half of the native and the non-native species were not found after being subjected to a water level fluctuation. From 2008 to 2015, only 27 native species always presented; however, not all of them were chosen as candidates for ecological restoration because of their decreased importance values. In contrast, the importance value of *Cynodon dactylon* increased over time, suggesting its high tolerance to long-term winter flooding. We concluded that riparian plants’ composition of the canyon landform area dramatically declined after long-term water level fluctuation and their presence was determined by the novel hydrological condition. Our results also suggested that *Cynodon dactylon* or its combination with other species (i.e. *Digitaria chrysoblephara*, *Setaria glauca*, *Setaria viridis*) is a better candidate for ecological restoration in the WLFZ.

## Introduction

Globally, there are over 13,000 dams higher than 30 m [1]. Dam building enhances human welfare, such as hydroelectric power generation and flood control, while simultaneously modifies the hydrological conditions of rivers. As a result, hydrological alteration has a dramatically negative (or positive) impact on riparian plant composition and diversity [2–5]. Riparian plants are a principal component of riparian ecosystems and play a vital role in the function of these ecosystems [6–8]. Therefore, riparian vegetation degradation resulting from hydrological alteration may lead to a series of ecological problems, including water eutrophication, soil degradation and erosion, geology disasters, and biodiversity loss [6–11]. As these problems have become global challenges [10], it is necessary to better understand the effect of hydrological change on riparian plants’ composition and to screen candidate plants for successful ecological restoration [2–5,12–14].

The China’s Three Gorges Dam (TGD), which is 185 m in height, was initiated in 1994. Its first impoundment was conducted in 2003, with a water level rising to 135 m, after which the water level rose to 156 m in 2006, 172 m in 2008 and 175 m in 2010. The Three Gorges Reservoir (TGR) formed when the water was raised to 175 m (the ultimate planned level) in 2010, with a riverbank surface area of approximately 1080 km^2^ [6–8]. To decrease sediment deposition and prolong the operational life of the TGR [13], water levels of the TGR varied by the anti-seasonal impoundment (the water level is highest (175 m) in winter and lowest (145 m) in summer), thereby producing a water level fluctuation zone (WLFZ) of approximately 350 km^2^ between the lowest and highest marks. The most unique characteristic in the hydrological regime between the TGR and other large reservoirs worldwide is the regular winter impoundment period that can be as long as half a year [13].

As expected, the results of short-term investigations in the WLFZ have shown that the number of riparian plants significantly declined under the influence of hydrological alteration [12,13,15–18]. However, there is little information for better understanding the long-term effects of water level fluctuation on riparian plants’ composition, especially in the WLFZ of the canyon landform area (a slope profile with a gradient greater than 25°) [12,18]. Additionally, ecological restoration is essential for solving ecological problems [6–11] in the WLFZ, and a fundamental aspect is the screening of candidate plants [14,19]. The unique characteristics of the hydrological regime in the TGR [13] suggested that candidate plants to be used for ecological restoration must be flood tolerant. Nonetheless, selection of plants based on their long-term adaptabilities to water level fluctuation is also important [13,14].

In this study, we attempted to answer the following questions: (1) How does long-term flooding affect riparian plant composition? (2) Which species are suitable for ecological restoration in the WLFZ? Accordingly, we analyzed the changes of riparian plants’ composition and richness in different life forms and calculated their importance values to assess their responses to the hydrological alteration and to screen candidate plants for ecological restoration in the WLFZ of the TGR. Here, we reported eight consecutive years of riparian plant assessment in the WLFZ of the canyon landform area from 2008 to 2015. The findings will enrich our knowledge of the effects of anti-seasonal impoundment on riparian plants and will be useful for long-term biodiversity research and ecological restoration in the WLFZ of the TGR.

## Materials and methods

Ethics statement: For our permanent plots, no specific permissions were required. Although the water level fluctuation zone of the Three Gorges Reservoir is required to protect, scientific research activities in this zone do not require specific permissions because they can provide better information for the ecological protection of the zone. The impact of the Three Gorges Project on the ecosystem is complex and therefore long-term monitoring in different fields is important. Also, the field study did not involve endangered or protected species.

### Study region

**Fig 1:**
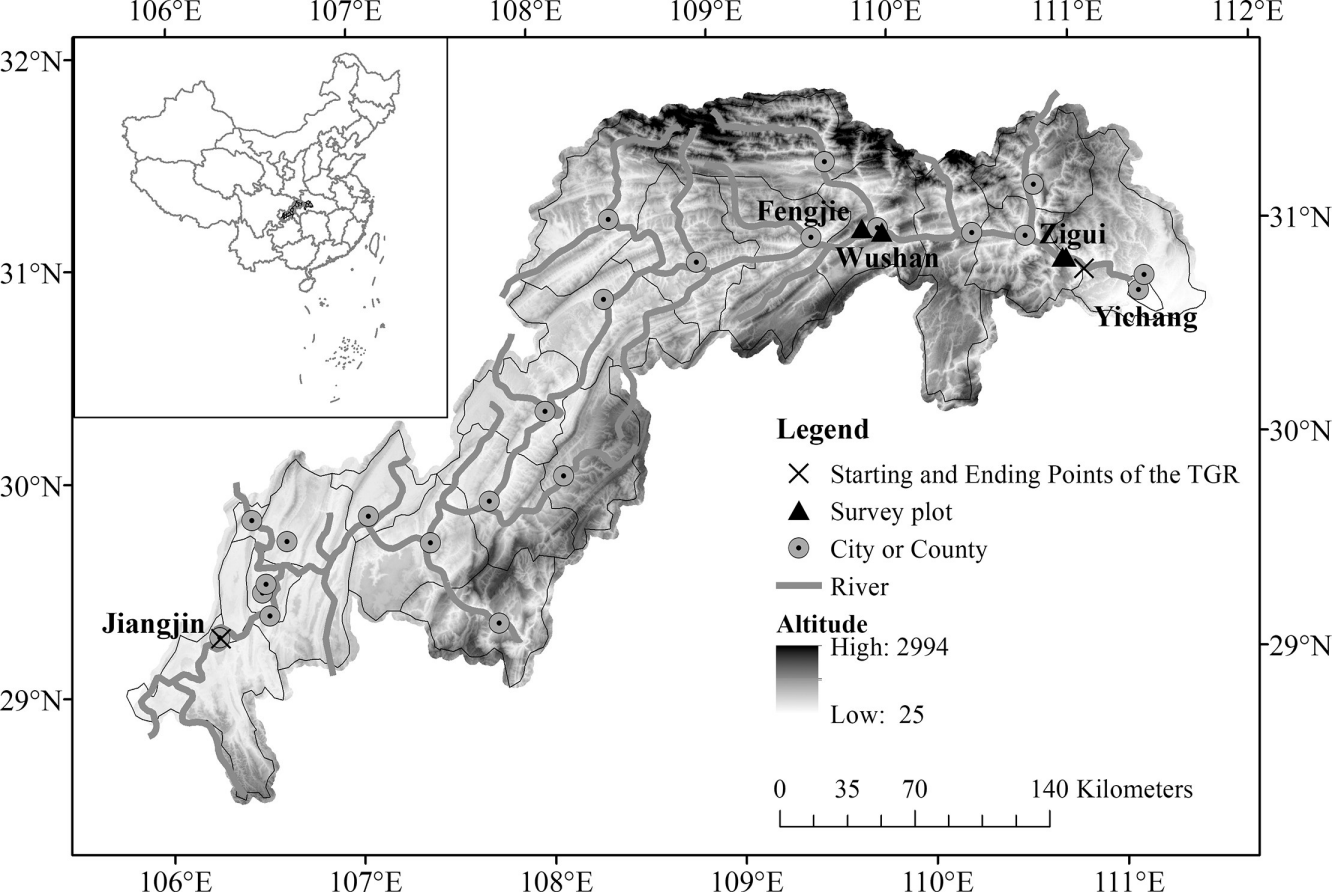
Locations of the study region and sampling plots in the water level fluctuation zone of China’s Three Gorges Reservoir. This map was created in ArcGIS 10.2 (http://www.esri.com).

The TGR stretches from Yichang City in Hubei Province to Jiangjin County in Chongqing Municipality (Fig 1). The WLFZ of the TGR was divided into three types based on geomorphologic characteristics: a slope profile with less than 15°, greater than 25°, and more than 45° [8]. Our study region (30°49′~31°16′ N, 109°27′~111°00′ E) is characterized by a slope profile with a gradient greater than 25°, which is typically distributed between Fengjie County and the dam and includes the deep valleys of tributaries (Fig 1), with a thin layer of bare soil (0–40 cm) [8]. In this region, ecological problems such as soil degradation and erosion, collapse, landslide, all caused by the loss of riparian vegetation [12,18] as well as the strong regional rainfall during the rainy season, have been confirmed [20–22].

The study region is characterized by subtropical humid monsoon climatic conditions with a mean annual temperature of 18.0–18.4 ^o^C. The mean annual precipitation is 1049.3–1100.0 mm and 70% of it falls from May to September. The ≥ 10.0 ^o^C accumulated temperature is 5723.6–5857.3 ^o^C. The annual frost-free period is 305 days. The zonal vegetation type is subtropical evergreen broad-leaved forest; however, due to the long-term interference of humans, the vegetation in the WLFZ before the water impoundment had been destroyed and replaced by a variety of secondary vegetation and artificial vegetation. Additionally, trees and large shrubs in the WLFZ on both sides of the reservoir were cleared prior to the first impoundment.

**Fig 2:**
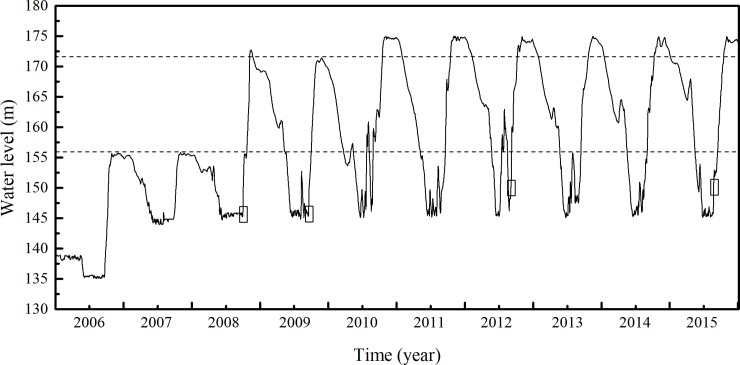
The water level fluctuation of the Three Gorges Reservoir during from 2006 to 2015. The data originated from the daily records of the Three Gorges Dam hydrology station (http://www.ctg.com.cn/). The dashed lines indicate water levels of 156 m and 172 m, respectively. The boxes indicate the periods of the field survey in 2008, 2009, 2012, and 2015.

In this study, study plots were located in Zigui County of Hubei Province and Wushan County of Chongqing Municipality (Fig 1) because of their typical geomorphologic characteristics [8,12,18]. Four permanent plots in the WLFZ (two in Zigui County and two in Wushan County) (Table 1, Fig 1) were used for riparian plant surveys in August 2008, 2009, 2012 and 2015. The surveys were conducted at an elevation zone of 156–172 m because this elevation zone experienced the same flooding frequency from 2008 to 2015 (Fig 2). More details about the four permanent plots are presented in Table 1.

**Table 1:** General characteristics of the four permanent plots in the water-level fluctuation zone of the Three Gorges Reservoir.

Plot	Coordinates	Slope	Soil thickness	Soil type	Pre-dam land use	Location
1	30°52.94'N, 110°54.35'E	28°	35cm	Yellow loan	Orchard	Confluence of the Yangtze River and Shamu Stream in Zigui County
2	30°53.05'N, 110°53.19'E	36°	40cm	Yellow loan	Forest land	Confluence of the Yangtze River and Lanling Stream in Zigui County
3	31°04.94'N, 109°54.13'E	32°	40cm	Yellow lime soil	Shrub land	Confluence of the Yangtze River and Daning River in Wushan County
4	31°03.56'N, 109°54.65'E	41°	35cm	Yellow lime soil	Shrub land	Main river of the Wushan section in of Yangtze River

### Hydrological regime characteristics

Before the impoundment of the TGR, the hydrological regime characteristics of the Yangtze River were similar to those of most other rivers in East Asia influenced by monsoon climate, with a high water level in summer and a low water level in winter [13]. Since the first impoundment of the TGR in 2003, however, the hydrological regime characteristics changed (Fig 2). The WLFZ was divided into three zones based on the flooding frequency: 145–156 m, 156–172 m, and 172–175 m. In general, the impoundment periods lasted more than eight months (mid-September to May), varying from year to year by a few days [13]. In addition, summer flooding in this study area increased to 160 m as a consequence of relatively heavy precipitation in 2010 and 2012 (Fig 2). Water levels in the survey period were 146 m in 2008, 146 m in 2009, 150 m in 2012 and 150 m in 2015.

### Field survey

Four permanent plots were established in the WLFZ of the canyon landform area in 2008 [12]. The length of the plot is 15 meters, and this line is parallel to the water surface of the TGR. The width of the plot varies with the slope, and this line is perpendicular to the water surface of the TGR. At each plot, we first placed seven transects at 2-m-high intervals from 156 m (the highest water level in winter of 2007) to 172 m (the highest water level in winter of 2008), and then divided the length into seven sections at 2-m intervals. Thus, there were 49 subplots in each plot. The plots were sampled in the same locations in 2008, 2009, 2012, and 2015. At each subplot, a plant survey was conducted within a square 1-m^2^ plot frame, placed at the centre of subplot to reduce the edge effect. Within each 1-m^2^ plot, the following variables were recorded for each species: abundance, average height, coverage and life form. For the tufted plants (or clonal plants), the abundance was determined by whether a single plant exists above the ground, and the continuity between their ramets or roots below the ground was not considered [18]. Plant coverage was estimated as the percent of the plot surface area for each species present [4]. Moreover, any species outside the plot but lying within the subplot were also recorded to obtain total plant richness [13]. For taxonomy and life form assignments, we referenced the database of the Flora of China (http://frps.eflora.cn/).

### Data analysis

To compare the adaptabilities and convergences of different riparian plants under the same or similar environment, four plots were merged into one analytical unit [12]. Therefore, there was only descriptive statistical analysis in this study. To understand the long-term responses of riparian plants’ composition to water level fluctuation of the TGR, we compiled data on the life tables of different plants from 2008 to 2015 (S1 Table). Riparian plants were classified into four life forms: trees, shrubs, annual herbs and perennial herbs. We also analyzed the temporal dynamics of richness (or the number of species) in different life forms, including native (first recorded in 2008) and non-native (first recorded in 2009, 2012, and 2015) riparian plants. In addition, the importance value of each species was employed as an indicator for assessing the impact of water level fluctuation and plant selection for ecological restoration. The importance value (*IV*) was calculated as *IV* = (*RC*+*RF*)/2, where *RC* and *RF* are the relative coverage and relative frequency, respectively [2,23]. Therefore, dominant plants (with importance values ranking within the top 10 in any survey from 2008 to 2015) and tolerant plants (native species always appearing in the WLFZ from 2008 to 2015) were listed alone.

## Results

### Changes in riparian plant composition

**Fig 3:**
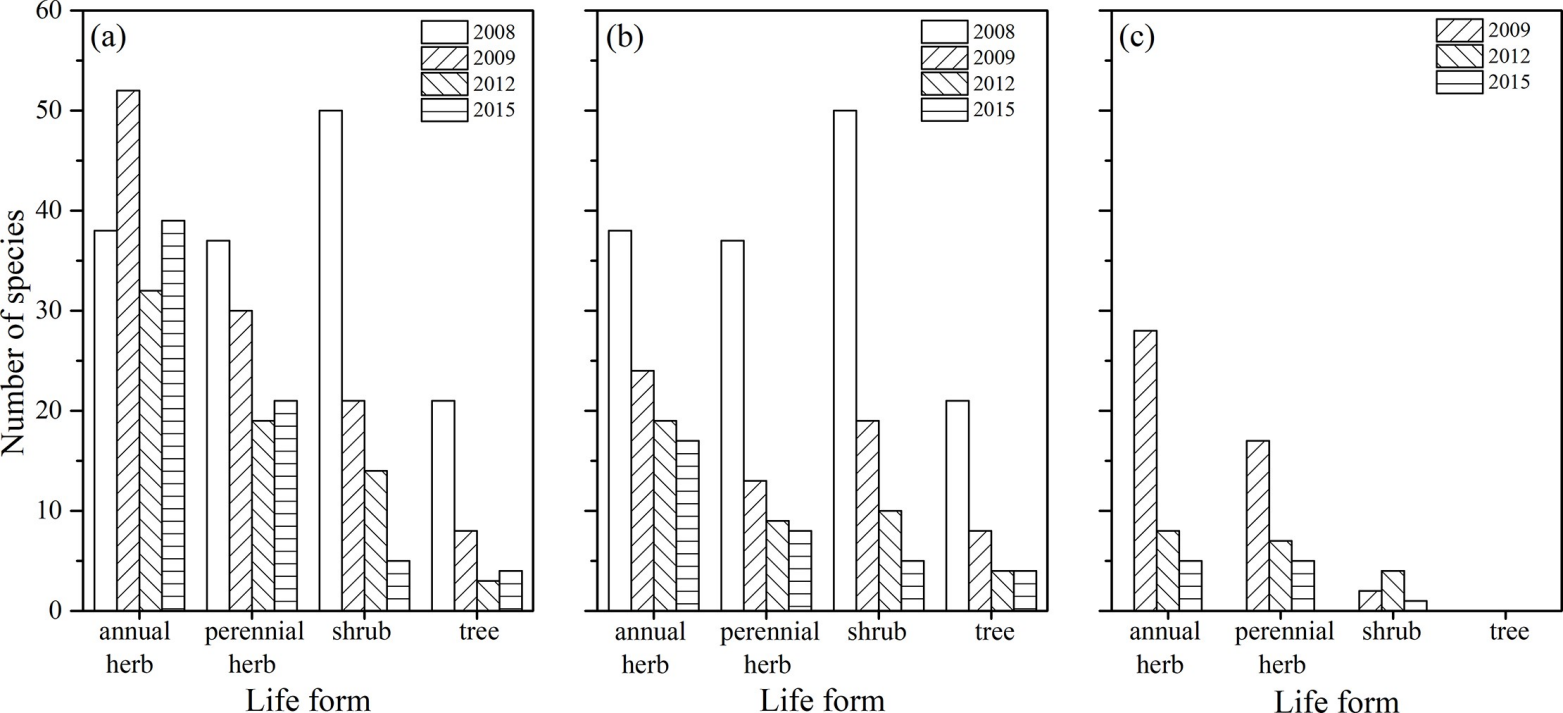
Variations in the number of total species (a), native species (b) and non-native species (c) in different years from 2008 to 2015 in the water level fluctuation zone of China’s Three Gorges Reservoir.

A total of 146 species in 2008, 110 in 2009, 68 in 2012, and 69 in 2015 were recorded (S1 Table, Fig 3A). Annual herbs fluctuated over time, whereas perennial herbs, shrubs and trees always exhibited a decreasing trend (Fig 3A). Annuals varied from 38 species in 2008 to 52 in 2009, 32 in 2012, and 39 in 2015.

These native species of different life forms always decreased with increasing survey time (Fig 3B). Over half of the native species in different life forms had disappeared by 2015. Indeed, 82 native species in 2009, 22 in 2012, and 8 in 2015 were not found. Moreover, almost all native species (37–65% for different life forms) were not recorded in 2009 (Fig 3B). Simultaneously, there were initial records of 47 non-native species in 2009, 19 in 2012, and 11 in 2015 (Fig 3C). These non-native species (77 species) mainly comprised herbs (41 annuals and 29 perennials) and were mainly found in 2009 (28 annuals and 17 perennials). In addition, most non-native species in 2009 and 2012 were found only once in the WLFZ (S1 Table).

Similarly, 56 families in 2008, 42 in 2009, 27 in 2012, and 27 in 2015 were recorded (S1 Table, Table 2). However, only one species was found for over half of the families in 2008, 2009, 2012, and 2015. For some large families with 6–9 species and over 10 species in 2008, changes into medium-sized families with 2–5 species or single species were observed for 2009, 2012, and 2015, including Leguminosae, Rosaceae, and Cyperaceae (S1 Table). Nonetheless, more than 10 species were always found for two families (Table 2), Asteraceae and Poaceae (8 species of Poaceae in 2012), during the survey period from 2008 to 2015. And these species were predominantly annuals (S1 Table).

**Table 2:** Variation in riparian plant families in 2008, 2009, 2012, and 2015 in the water level fluctuation zone of China’s Three Gorges Reservoir.

Survey period	Families with the following number of species				Total
	1 species	2–5 species	6–9 species	≥10 species	
2008	36	14	2	4	56
2009	27	11	2	2	42
2012	16	8	2	1	27
2015	17	7	1	2	27

### Importance values of dominant and tolerant riparian plants

The dominant plants were shrubs in 2008 while annual herbs in 2009, 2012 and 2015 (Fig 3A, Table 3). These dominant plants were mainly the native species and had variable importance values (Table 3). In 2008, plants with an importance value > 10 were not found. In 2009, the plants of note (IV>10) were *Setaria viridis* (10.78) and *Bidens pilosa* (10.65). *Digitaria chrysoblephara* (17.60), *Setaria viridis* (14.40), *Bidens pilosa* (12.96), *Cynodon dactylon* (11.43) and *Cyperus iria* (11.08) were notable in 2012. In 2015, importance values greater than 10 were found for *Cynodon dactylon* (30.55) and *Setaria viridis* (10.98).

**Table 3:** Importance values of plants ranked within the top 10 in 2008, 2009, 2012 and 2015 in the water level fluctuation zone of China’s Three Gorges Reservoir.

Species	2008	2009	2012	2015	Source
***Artemisia carvifolia***	2.10	6.21	-	-	native
***Bidens pilosa***	-	10.65	12.96	5.63	native
***Conyza bonariensis***	-	2.88	-	-	non-native
***Crassocephalum crepidioides***	-	5.82	-	-	native
***Cynodon dactylon***	-	-	11.43	30.55	non-native
***Cyperus iria***	-	-	11.08	2.64	native
***Cyperus rotundus***	-	5.66	-	3.09	non-native
***Digitaria chrysoblephara***	-	-	17.60	8.51	native
***Digitaria sanguinalis***	-	5.16	-	-	native
***Echinochloa crusgalli***	-	-	-	5.97	native
***Eclipta prostrata***	-	3.10	-	3.22	native
***Erigeron acer***	-	-	3.00	-	non-native
***Ficus tikoua***	1.82	-	-	-	native
***Heteropogon contortus***	-	-	2.07	-	native
***Koelreuteria bipinnata***	2.15	-	-	-	native
***Mallotus apelta***	2.41	-	-	-	native
***Miscanthus floridulus***	4.22	-	-	-	native
***Miscanthus sinensis***	3.77	-	-	-	native
***Polygonum orientale***	-	-	2.23	-	non-native
***Quercus variabilis***	4.29	-	-	-	native
***Rhus chinensis***	3.57	-	-	-	native
***Saccharum arundinaceum***	3.31	-	-	-	native
***Setaria glauca***	-	-	-	7.80	native
***Setaria palmifolia***	-	3.82	-	-	native
***Setaria viridis***	-	10.78	14.40	10.98	native
***Solanum nigrum***	-	5.19	2.71	-	native
***Vitex negundo***	2.62	-	-	-	native
***Xanthium sibiricum***	-	-	4.87	9.42	non-native

“-” indicates that the importance value was not available for a survey year.

A total of 27 native species (Table 4) showed stronger tolerance to water level fluctuation from 2008 to 2015 in the WLFZ, including 7 woody plants and 20 herbaceous plants (S1 Table). However, the importance values of most tolerant species gradually declined from 2008 to 2015. In contrast, these plants, such as *Bidens pilosa*, *Digitaria chrysoblephara*, *Setaria glauca* and *Setaria viridis*, exhibited increasing importance values from 2008 to 2015.

**Table 4:** Importance values of riparian plants that always appeared in 2008, 2009, 2012 and 2015 in the water level fluctuation zone of China’s Three Gorges Reservoir.

Species	2008	2009	2012	2015
***Acalypha australis***	0.47	0.90	0.63	0.95
***Artemisia carvifolia***	2.10	6.21	0.80	0.13
***Artemisia lancea***	0.27	0.13	0.16	0.05
***Bidens pilosa***	0.63	10.65	12.96	5.63
***Boehmeria nivea***	0.76	0.50	0.18	0.05
***Coriaria nepalensis***	1.43	0.51	0.33	0.05
***Cyperus iria***	0.57	0.37	11.08	2.65
***Dendranthema indicum***	0.85	1.61	0.60	0.07
***Digitaria chrysoblephara***	0.21	1.92	17.60	8.51
***Echinochloa crusgalli***	0.12	1.03	0.66	5.97
***Eclipta prostrata***	0.30	3.11	1.28	3.22
***Eupatorium lindleyanum***	0.53	0.13	0.08	0.05
***Glochidion puberum***	0.90	0.62	0.71	0.15
***Heteropogon contortus***	0.50	0.13	2.07	+
***Indigofera pseudotinctoria***	1.24	0.86	0.16	0.06
***Ixeris polycephala***	0.28	0.25	0.20	0.07
***Kalimeris indica***	0.94	0.27	0.39	0.05
***Koelreuteria bipinnata***	2.15	0.22	0.25	+
***Oxalis corniculata***	1.01	1.11	1.70	0.37
***Phyllanthus urinaria***	1.09	0.74	0.16	0.20
***Pogonatherum crinitum***	0.11	+	0.09	+
***Rhus chinensis***	3.57	1.60	0.28	0.05
***Sapium sebiferum***	1.57	1.72	0.32	0.05
***Setaria glauca***	0.12	0.13	0.80	7.80
***Setaria viridis***	1.22	10.79	14.40	10.94
***Solanum nigrum***	0.23	5.20	2.71	0.65
***Vitex negundo***	2.62	2.01	0.34	+

“+” indicates that the species was not found in the 1-m^2^ plot but was found in the subplot.

## Discussions

### Ecological responses of riparian plants to water level fluctuation

The riparian plants in the study area exhibited different levels of ecological responses to the water level fluctuation of the TGR (S1 Table) as a result of differences in the time, duration, depth and frequency of flooding [2–5,12,13,15–18]. We found that the number of riparian plants (i.e., native species) substantially declined from 2008 to 2015 (Fig 3A and 3B) due to long-term winter impoundment (Fig 2). In general, plants have stable structures and physiology to adapt to environmental conditions during long-term historical evolution and therefore can modulate their adaptabilities to environmental changes with regard to morphological alterations or physiological processes [24]. In this study, however, the formation time of the WLFZ in the elevation zone of 156–172 m was sufficiently short (8 years) that the adaptabilities of riparian plants were limited because of instantaneous and accidental flooding [18]. Additionally, most riparian plants do not possess the structure and function to acclimate the extreme flooding conditions [15,16,25–27]. These two reasons led to the death or disappearance of a large number of riparian plants from 2008 to 2015 (Fig 3A) and a significant decrease of native species in 2009 (Fig 3B), which is in line with previous studies [12,15,17,18]. Nonetheless, a number of the non-native plants were observed in the WLFZ from 2009 to 2015 (Fig 3C) due to successful seed germination from the soil seed bank after winter impoundment [23,28–30] and/or from the upper reaches of the Yangtze River as well as the terrestrial ecosystem that was not flooded by the impoundment of the TGR. One possible reason for the loss of most non-native species (S1 Table) is that they lacked of adaptabilities to winter flooding. It is also possible that these non-native species had no advantages in resource competition compared to native species [31].

We found that riparian species of two families, Asteraceae and Poaceae, exhibited stronger tolerance to the water level fluctuations from 2008 to 2015 (S1 Table, Tables 2–4) due to their biological characteristics (e.g., short growth period, small individual plant and seed size, large number of seeds) and origin as well as to their greater capacity to adapt to environmental change [12,18,24]. For example, the caryopses of Poaceae plants, such as *Digitaria chrysoblephara*, *Setaria glauca*, and *Setaria viridis*, have special appendages that can serve to increase their distribution ranges. In addition, some plants such as *Cynodon dactylon* mainly rely on belowground rhizomes and stolons to reproduce; thus, they can occupy a habitat by rapidly reproducing during a growth period [31–35]. Asteraceae plants such as *Bidens pilosa* and *Dendranthema indicum* have smaller seeds but more fruit, and their seeds have appendages, such as cresteds, bristles and hooks, to facilitate long-distance dispersal [12,18,24]. Furthermore, seeds of Poaceae and Asteranceae plants have longer vitalities and can survive in the long-term flooding environment [23]; in a stressful environment, these plants will also complete their life cycle over a relatively short period [12]. At the same time, our surveys also found that some shrubs and trees (i.e., *Coriaria nepalensis*, *Glochidion puberum*, *Rhus chinensis*, and *Koelreuteria bipinnata*) (S1 Table) existed in the form of annual seedlings because of their stronger germination abilities and their growth cycle in line with the flooding-drying habitats in the WLFZ [16,18].

### Plant selection for ecological restoration

Ecological restoration is essential to solve the ecological problems in the WLFZ, and a fundamental aspect is the selection of suitable plants [14,19]. To achieve this objective, laboratory experiments simulating the altered environment of the TGR and planting experiments in the WLFZ have been conducted [32–45]. Species including *Arundinella anonmala* [36], *Cynodon dactylon* and *Hemarthria altissima* [32], *Cyperus rotundu* [37], *Salix variegates* [36,38], and *Distylium chinense* [39] in laboratory experiments and *Cyperus rotundu* [40], *Cynodon dactylon* [33–35], *Vetiveria zizanioides* [41], *Saccharum spontaneum* [42], *Morus alba* [43], *Taxodium ascendens* [44], and *Taxodium distichum* [45] in planting experiments have been suggested as potential candidates. However, most species identified in laboratory experiments cannot persist well in the hydrological regime characteristics of the TGR [13,14] because the practical water-level scheduling is different from the simulated water-level scheme with regards to flooding rhythm, duration and depth [14]. Additionally, the short-term results from the planting experiments (less than 3 years) at the initial period of the water level fluctuation have great uncertainties because of the severe variation in environmental factors [12].

One previous study suggested that the candidates for ecological restoration must be flood tolerant [13]. In this study, 27 native species exhibited strong resistance to long-term water level fluctuations from 2008 to 2015 (Table 4), though most of these native species may disappear from the WLFZ in the future because of their decreased importance values (Table 4). Although plants such as *Bidens pilosa*, *Digitaria chrysoblephara*, *Echinochloa crusgalli*, *Setaria glauca* and *Setaria viridis* exhibited better adaptabilities to water level fluctuation (Table 4), they were not found to be suitable candidates for ecological restoration. One possible reason is that their litter releases more nitrogen and phosphorus under flooding conditions than does that of other plants such as *Cynodon dactylon*, *Dendranthema indicum* and *Hemarthria altissima* [46,47], thereby increasing the risk of eutrophication of the soil and the reservoir. In fact, *Cynodon dactylon*, a non-native species in this study, displayed the highest tolerance to water level fluctuation in the WLFZ due to its increasing importance value from 2008 to 2015 (Table 3) [13], which further confirmed the results of previous laboratory [32] and planting [33–35] experiments. Therefore, *Cynodon dactylon* is a better candidate plant for ecological restoration in the WLFZ of the TGR. Of course, in the early stages of riparian ecosystem succession, annual plants still dominate, indicating some plants (Tables 3 and 4) can be used as candidates if effectively alleviating their pollution risk.

### Differences between short- and long-term surveys

The anti-seasonal impoundment of the TGR differs from that of other reservoirs around the world [13], and accordingly, this riparian ecosystem has drawn attention from ecologists and governments. Based on short-term surveys from 2008 to 2010 [12,13,15–18], the number of riparian plants decreased significantly after winter impoundment, which was in accordance with the findings of the long-term survey from 2008 to 2015 (Fig 3A and 3B). Also, we found that the speed of reduction decreased over flood frequency (Fig 3A and 3B). However, the composition of riparian plants did not differ between long-term (S1 Table) and short-term [12,18] investigations. For example, dominant plants included *Cyperus rotundus*, *Gynura crepidioides* and *Artemisia apiacea* in short-term survey [12] while they were not found in long-term survey (Table 3). Moreover, the finding that most of the non-native species (Fig 3C) cannot form dominant plant communities (Table 3) was consistent with a previous study [15]. Although some woody plants (S1 Table) existed as annual seedlings in the WLFZ from 2008 to 2015, we suggested that these species were not suitable candidates for ecological restoration because of their poor adaptabilities to long-term flooding of the TGR (over 10 years, unpublished data). This result was in contrast with that of analyses after controlled winter flooding [16,43–45], which showed that some woody plants such as *Salix matsudana*, *Morus alba*, *Taxodium* spp. and *Distylium chinensis* might be considered candidates for riparian protection forests in the WLFZ. Based on our results, we suggested that *Cynodon dactylon* or its combination with other annual plants (i.e. *Digitaria chrysoblephara*, *Setaria glauca*, *Setaria viridis*, Tables 3 and 4) is suitable for ecological restoration, which agreed with previous studies [13,35].

## Conclusions

In this study, we found that riparian plants in the WLFZ significantly decreased over time from 2008 to 2015, which may lead to serious ecological problems, such as ecological vulnerability, soil erosion, landslides, water pollution and frequent pests and diseases. Therefore, riparian vegetation should be restored and rebuilt using plants that are tolerant to long-term water level fluctuations. Although 27 tolerant native plants were detected repeatedly from 2008 to 2015, not all of them will adapt to the future long-term water level fluctuation. Indeed, our results from long-term monitoring indicate that *Cynodon dactylon* or its combination with other annual plants (i.e. *Digitaria chrysoblephara*, *Setaria glauca*, *Setaria viridis*) is better candidate for ecological restoration in the WLFZ of the TGR because of its strong ecological adaptability to water level fluctuation.

## Supporting information

S1 Table List of riparian plants in 2008, 2009, 2012 and 2015 in the water level fluctuation zone of canyon landform area of China’s Three Gorges Reservoir. Ah: Annual herb; Ph: Perennial herb; S: Shrub; T: Tree. + Indicated appeared species in any survey year (2008–2015). a The genera number within each family. b The species number within each family.
(DOC)
