# Supplementary material for: Long-term responses of riparian plants’ composition to water level fluctuation in China's Three Gorges Reservoir
Source: PLoS One. 2018 Nov 28;13(11):e0207689. doi: 10.1371/journal.pone.0207689 (PMC6261589; doi:10.1371/journal.pone.0207689)
Supplement: S1 Table — Ah: Annual herb; Ph: Perennial herb; S: Shrub; T: Tree. + Indicated appeared species in any survey year (2008–2015). a The genera number within each family. b The species number within each family. (DOC) [file pone.0207689.s001.doc]

**S1 Table. List of riparian plants in 2008, 2009, 2012 and 2015 in the water level fluctuation zone of canyon landform area of China’s Three Gorges Reservoir.**

| 2008 | 2009 | 2012 | 2015 | Life form |
| --- | --- | --- | --- | --- |
| 1 Acanthaceae (1^a^:1^b^) | 1 Acanthaceae (1^a^:1^b^) |  |  |  |
| *Rhus chinensis* | + |  |  | Ah |
| 2 Anacardiaceae (2:3) | 2 Anacardiaceae (1:1) | 2 Anacardiaceae (1^a^:1^b^) | 2 Anacardiaceae (1^a^:1^b^) |  |
| *Rhus chinensis* | + | + | + | T |
| *Toxicodendron succedaneum* |  |  |  | T |
| *Toxicodendron sylvestre* |  |  |  | T |
| 3 Aquifoliaceae (1:1) |  |  |  |  |
| *Ilex cornuta* |  |  |  | S |
| 4 Araliaceae (1:1) |  |  |  |  |
| *Aralia chinensis* |  |  |  | S |
| 5 Asteraceae (15:20) | 5 Asteraceae (18:19) | 5 Asteraceae (16:20) | 5 Asteraceae (13:16) |  |
| *Artemisia carvifolia* | + | + | + | Ah |
| *Artemisia indices* |  |  |  | Ph |
| *Artemisia lancea* | + | + | + | Ph |
|  |  | *Artemisia annua* | + | Ah |
|  |  | *Artemisia capillaris* |  | Ah |
| *Aster ageratoides* |  |  |  | Ph |
| *Aster subulatus* |  |  |  | Ph |
| *Bidens pilosa* | + | + | + | Ah |
| *Carpesium abrotanoides* |  | + |  | Ph |
| *Carpesium cernuum* |  |  |  | Ph |
| *Conyza canadensis* |  |  |  | Ah |
| *Conyza sumatrensis* |  |  |  | Ah |
|  | *Conyza bonariensis* |  |  | Ah |
| *Crassocephalum crepidioides* | + | + |  | Ah |
| *Dendranthema indicum* | + | + | + | Ph |
| *Eclipta prostrata* | + | + | + | Ah |
| *Eupatorium lindleyanum* | + | + | + | Ph |
| *Ixeris polycephala* | + | + | + | Ah |
| *Kalimeris indica* | + | + | + | Ah |
| *Pterocypsela formosana* | + |  |  | Ah |
| *Solidago decurrens* |  |  |  | Ph |
| *Sonchus oleraceus* | + | + |  | Ah |
| *Youngia japonica* | + | + |  | Ah |
|  | *Emilia sonchifolia* |  |  | Ah |
|  | *Gnaphalium affine* | + | + | Ah |
|  | *Hemistepta lyrata* | + | + | Ah |
|  | *Senecio scandens* |  |  | Ph |
|  | *Siegesbeckia orientalis* |  |  | Ah |
|  | *Xanthium sibiricum* | + | + | Ah |
|  |  | *Erigeron acer* | + | Ah |
|  |  | *Erigeron annuus* | + | Ah |
|  |  | *Lagedium sibiricum* | + | Ph |
|  |  |  | *Leontopodium leontopodioides* | Ph |
| 6 Boraginaceae (1:1) | 6 Boraginaceae (2:2) |  | 6 Boraginaceae (2:2) |  |
| *Ehretia macrophylla* |  |  |  | T |
|  | *Bothriospermum tenellum* |  | + | Ah |
|  | *Cynoglossum amabile* |  |  | Ph |
|  |  |  | *Trigonotis peduncularis* | Ah |
| 7 Caprifoliaceae (3:3) | 7 Caprifoliaceae (1:1) |  |  |  |
| *Abelia chinensis* | + |  |  | S |
| *Lonicera mucronata* |  |  |  | S |
| *Viburnum utile* |  |  |  | S |
| 8 Celastraceae (1:1) | 8 Celastraceae (1:1) |  |  |  |
| *Maytenus variabilis* | + |  |  | S |
| 9 Convolvulaceae (1:1) | 9 Convolvulaceae (1:1) | 9 Convolvulaceae (2:2) | 9 Convolvulaceae (1:1) |  |
| *Calystegia hederacea* |  | + | + | Ah |
|  | *Convolvulus arvensis* |  |  | Ph |
|  |  | *Dichondra repens* |  | Ph |
| 10 Coriariaceae (1:1) | 10 Coriariaceae (1:1) | 10 Coriariaceae (1:1) | 10 Coriariaceae (1:1) |  |
| *Coriaria nepalensis* | + | + | + | S |
| 11 Cyperaceae (4:8) | 11 Cyperaceae (2:5) | 11 Cyperaceae (1:1) | 11 Cyperaceae (1:2) |  |
| *Carex brunnea* | + |  |  | Ph |
| *Carex chlorostachys* |  |  |  | Ph |
| *Carex cruciata* |  |  |  | Ph |
| *Carex rubrobrunnea var. taliensis* |  |  |  | Ph |
|  | *Carex lancifolia* |  |  | Ph |
|  | *Carex phyllocephala* |  |  | Ph |
| *Cyperus iria* | + | + | + | Ah |
| *Cyperus microiria* |  |  |  | Ah |
|  | *Cyperus rotundus* |  | + | Ph |
| *Eriophorum comosum* |  |  |  | Ph |
| *Scleria hookeriana* |  |  |  | Ah |
| 12 Dioscoreaceae (1:2) |  |  |  |  |
| *Dioscorea bulbifera* |  |  |  | Ph |
| *Dioscorea zingiberensis* |  |  |  | Ph |
| 13 Dryopteridaceae (1:1) |  |  |  |  |
| *Dryopteris championii* |  |  |  | Ph |
| 14 Ebenaceae (1:1) | 14 Ebenaceae (1:1) |  |  |  |
| *Diospyros lotus* | + |  |  | T |
| 15 Ericaceae (1:1) | 15 Ericaceae (1:1) |  |  |  |
| *Rhododendron simsii* |  |  |  | S |
|  | *Vaccinium mandarinorum* |  |  | S |
| 16 Euphorbiaceae (8:8) | 16 Euphorbiaceae (7:7) | 16 Euphorbiaceae (5:6) | 16 Euphorbiaceae (6:8) |  |
| *Acalypha australis* | + | + | + | Ah |
| *Discocleidion rafuescens* | + |  | + | S |
| *Glochidion puberum* | + | + | + | S |
| *Mallotus apelta* | + |  |  | T |
| *Phyllanthus urinaria* | + | + | + | Ah |
|  |  |  | *Phyllanthus ussuriensis* | Ah |
| *Sapium sebiferum* | + | + | + | T |
| *Speranskia cantonensis* |  |  |  | Ah |
| *Vernicia fordii* |  |  |  | T |
|  | *Euphorbia hirta* | + |  | Ah |
|  |  | *Euphorbia pekinensis* | + | Ph |
|  |  |  | *Euphorbia humifusa* | Ah |
| 17 Fagaceae (2:2) | 17 Fagaceae (1:1) |  |  |  |
| *Castanea mollissima* |  |  |  | T |
| *Quercus variabilis* | + |  |  | T |
| 18 Flacourtiaceae (1:1) |  |  |  |  |
| *Xylosma racemosum* |  |  |  | S |
| 19 Guttiferae (1:1) |  | 19 Guttiferae (1:1) |  |  |
| *Hypericum monogynum* |  | + |  | S |
| 20 Hamamelidaceae (1:1) |  |  |  |  |
| *Loropetalum chinense* |  |  |  | T |
| 21 Juglandaceae (1:1) |  |  |  |  |
| *Platycarya strobilacea* |  |  |  | T |
| 22 Labiatae (2:2) | 22 Labiatae (3:3) | 22 Labiatae (3:3) | 22 Labiatae (1:1) |  |
| *Perilla frutescens* |  | + |  | Ah |
| *Pogostemon auricularius* | + |  |  | Ah |
|  | *Salvia plebeia* |  | + | Ah |
|  | *Scutellaria franchetiana* | + |  | Ph |
|  |  | *Leonurus artemisia* |  | Ah |
| 23 Lardizabalaceae (1:1) |  |  |  |  |
| *Akebia trifoliata* |  |  |  | S |
| 24 Lauraceae (2:3) | 24 Lauraceae (1:1) |  |  |  |
| *Lindera communis* |  |  |  | S |
| *Lindera glauca* | + |  |  | S |
| *Litsea mollis* |  |  |  | S |
| 25 Leguminosae (8:11) | 25 Leguminosae (4:5) | 25 Leguminosae (2:2) | 25 Leguminosae (1:1) |  |
| *Campylotropis macrocarpa* |  |  |  | T |
| *Dalbergia hupeana* |  |  |  | T |
| *Gymnocladus chinensis* |  |  |  | T |
| *Indigofera pseudotinctoria* | + | + | + | S |
| *Kummerowia stipulacea* |  |  |  | Ah |
| *Lespedeza chinensis* |  |  |  | S |
| *Lespedeza cuneata* | + |  |  | S |
| *Lespedeza formosa* | + | + |  | S |
| *Mucuna sempervirens* | + |  |  | S |
| *Rhynchosia dielsii* |  |  |  | Ah |
| *Rhynchosia volubilis* |  |  |  | Ah |
|  | *Sesbania cannabina* |  |  | Ah |
| 26 Liliaceae (3:3) |  |  |  |  |
| *Lilium brownii var. viridulum* |  |  |  | Ah |
| *Liriope spicata* |  |  |  | Ph |
| *Ophiopogon japonicus* |  |  |  | Ph |
| 27 Lygodiaceae (1:1) | 27 Lygodiaceae (1:1) | 27 Lygodiaceae (1:1) |  |  |
| *Lygodium japonicum* | + | + |  | Ph |
| 28 Meliaceae (1:1) |  |  |  |  |
| *Toona sinensis* |  |  |  | T |
| 29 Moraceae (2:2) | 29 Moraceae (2:2) |  | 29 Moraceae (2:2) |  |
| *Broussonetia papyifera* | + |  | + | T |
| *Ficus tikoua* | + |  | + | Ph |
| 30 Myrsinaceae (1:1) | 30 Myrsinaceae (1:1) |  |  |  |
| *Myrsine africana* | + |  |  | S |
| 31 Oleaceae (2:2) |  | 31 Oleaceae (1:1) |  |  |
| *Jasminum floridum* |  |  |  | S |
|  |  | *Jasminum mesnyi* |  | S |
| *Ligustrum quihoui* |  |  |  | S |
| 32 Oxalidaceae (1:1) | 32 Oxalidaceae (1:1) | 32 Oxalidaceae (1:1) | 32 Oxalidaceae (1:1) |  |
| *Oxalis corniculata* | + | + | + | Ah |
| 33 Palmae (1:1) |  |  |  |  |
| *Trachycarpus fortunei* |  |  |  | S |
| 34 Phytolaccaceae (1:1) | 34 Phytolaccaceae (1:1) | 34 Phytolaccaceae (1:1) |  |  |
| *Phytolacca acinosa* | + | + |  | Ph |
| 35 Poaceae (13:18) | 35 Poaceae (12:16) | 35 Poaceae (7:8) | 35 Poaceae (9:11) |  |
| *Arthraxon hispidus* | + |  | + | Ah |
| *Arthraxon lanceolatus* |  |  |  | Ph |
| *Brachiaria villosa* |  |  | + | Ah |
| *Capillipedium assimile* |  |  |  | Ph |
|  | *Capillipedium parviflorum* |  |  | Ph |
| *Digitaria chrysoblephara* | + | + | + | Ah |
|  | *Digitaria sanguinalis* |  | + | Ah |
| *Echinochloa crusgalli* | + | + | + | Ah |
| *Eulaliopsis binata* |  |  |  | Ph |
| *Heteropogon contortus* | + | + | + | Ph |
| *Imperata cylindrica* |  |  |  | Ph |
| *Miscanthus floridulus* |  |  |  | Ph |
| *Miscanthus sinensis* |  |  |  | Ph |
| *Neyraudia reynaudiana* |  |  |  | Ph |
| *Pogonatherum crinitum* | + | + | + | Ph |
| *Saccharum arundinaceum* | + |  |  | Ph |
| *Setaria faberii* | + |  |  | Ah |
| *Setaria glauca* | + | + | + | Ah |
| *Setaria palmifolia* | + |  |  | Ah |
| *Setaria viridis* | + | + | + | Ah |
|  | *Eleusine indica* |  | + | Ah |
|  | *Hemarthria altissima* |  |  | Ph |
|  | *Leptochloa panicea* |  |  | Ah |
|  | *Oplismenus undulatifolius* |  |  | Ah |
|  |  | *Cynodon dactylon* | + | Ph |
|  |  | *Sorghum sudanense* |  | Ah |
| 36 Polygalaceae (1:1) |  |  |  |  |
| *Polygala sibirica* |  |  |  | Ph |
| 37 Primulaceae (1:1) |  |  |  |  |
| *Lysimachia congestiflora* |  |  |  | Ah |
| 38 Pteridaceae (1:1) | 38 Pteridaceae (1:1) |  | 39 Pteridaceae (1:3) |  |
| *Pteris vittata* | + |  | + | Ph |
|  |  |  | *Pteris cretica var. nervosa* | Ph |
|  |  |  | *Pteris multifida* | Ph |
| 39 Rhamnaceae (2:4) |  |  |  |  |
| *Rhamnus crenata* |  |  |  | S |
| *Rhamnus heterophylla* |  |  |  | S |
| *Rhamnus rosthornii* |  |  |  | S |
| *Sageretia thea* |  |  |  | S |
| 40 Rosaceae (4:10) | 40 Rosaceae (3:7) | 40 Rosaceae (2:3) |  |  |
| *Pyracantha fortuneana* |  |  |  | S |
| *Rosa cymosa* | + |  |  | S |
| *Rosa henryi* |  |  |  | S |
| *Rosa laevigata* | + |  |  | S |
| *Rosa multiflora* |  | + |  | S |
| *Rubus corchorifolius* | + | + |  | S |
| *Rubus coreanus* | + |  |  | S |
| *Rubus innominatus* | + |  |  | S |
| *Rubus parvifolius* | + | + |  | S |
| *Spiraea chinensis* |  |  |  | S |
|  | *Duchesnea indica* |  |  | Ph |
| 41 Rubiaceae (2:2) | 41 Rubiaceae (1:1) |  |  |  |
| *Mussaenda pubescens* |  |  |  | S |
| *Paederia scandens* | + |  |  | Ph |
| 42 Rutaceae (1:1) |  |  |  |  |
| *Zanthoxylum stenophyllum* |  |  |  | S |
| 43 Sapindaceae (1:1) | 43 Sapindaceae (1:1) | 43 Sapindaceae (1:1) | 43 Sapindaceae (1:1) |  |
| *Koelreuteria bipinnata* | + | + | + | T |
| 44 Scrophulariaceae (1:1) | 44 Scrophulariaceae (2:2) |  | 44 Scrophulariaceae (1:1) |  |
| *Paulownia fortunei* |  |  |  | T |
|  | *Mazus japonicus* |  | + | Ah |
|  | *Mimulus tenellus* |  |  | Ph |
| 45 Selaginellaceae (1:1) |  |  |  |  |
| *Selaginella nipponica* |  |  |  | Ph |
| 46 Smilacaceae (2:2) |  |  |  |  |
| *Heterosmilax japonica* |  |  |  | S |
| *Smilax china* |  |  |  | S |
| 47 Solanaceae (2:2) | 47 Solanaceae (2:3) | 47 Solanaceae (1:2) | 47 Solanaceae (2:2) |  |
| *Physalis angulata* | + |  |  | Ah |
| *Solanum nigrum* | + | + | + | Ah |
|  | *Solanum lyratum* | + |  | Ph |
|  |  |  | *Darura stramonium* | Ah |
| 48 Symplocaceae (1:1) |  |  |  |  |
| *Symplocos paniculata* |  |  |  | S |
| 49 Taxodiaceae (1:1) |  |  |  |  |
| *Cunninghamia lanceolata* |  |  |  | T |
| 50 Theaceae (1:1) |  |  |  |  |
| *Camellia sinensis* |  |  |  | S |
| 51 Thelypteridaceae (1:1) |  |  |  |  |
| *Cyclosorus acuminatus* |  |  |  | Ph |
| 52 Thymelaeaceae (1:1) |  |  |  |  |
| *Wikstroemia micrantha* |  |  |  | S |
| 53 Ulmaceae (1:1) | 53 Ulmaceae (1:1) |  |  |  |
| *Trema levigata* | + |  |  | T |
| 54 Urticaceae (1:1) | 54 Urticaceae (2:3) | 54 Urticaceae (2:2) | 54 Urticaceae (2:3) |  |
| *Boehmeria nivea* | + | + | + | Ph |
|  | *Boehmeria macrophylla* |  | + | P Ph |
|  | *Pouzolzia zeylanica* |  | + | Ph |
|  |  | *Urtica fissa* |  | Ph |
| 55 Verbenaceae (1:1) | 55 Verbenaceae (1:1) | 55 Verbenaceae (1:1) | 55 Verbenaceae (1:1) |  |
| *Vitex negundo* | + | + | + | S |
| 56 Vitaceae (2:3) | 56 Vitaceae (1:1) | 56 Vitaceae (2:2) |  |  |
| *Ampelopsis delavayana* |  |  |  | S |
| *Vitis flexuosa* |  |  |  | S |
| *Vitis heyneana* | + | + |  | S |
|  |  | *Parthenocissus quinquefolia* |  | S |
|  | 57 Aizoaceae (1:1) |  |  |  |
|  | *Mollugo stricta* |  |  | Ah |
|  | 58 Amaranthaceae (3:4) |  | 58 Amaranthaceae (2:2) |  |
|  | *Alternanthera philoxeroides* |  |  | Ph |
|  | *Amaranthus spinosus* |  |  | Ah |
|  | *Amaranthus viridis* |  | + | Ah |
|  | *Celosia argentea* |  | + | Ah |
|  | 59 Buddlejaceae (1:1) |  |  |  |
|  | *Buddleja lindleyana* |  |  | S |
|  | 60 Commelinaceae (1:1) |  |  |  |
|  | *Cyanotis arachnoidea* |  |  | Ph |
|  | 61 Cucurbitaceae (1:1) |  |  |  |
|  | *Melothria ndica* |  |  | Ah |
|  | 62 Lentibulariaceae (1:1) |  |  |  |
|  | *Utricularia bifida* |  |  | Ah |
|  | 63 Malvaceae (2:2) |  | 63 Malvaceae (1:1) |  |
|  | *Abutilon theophrasti* |  | + | Ah |
|  | *Urena lobata* |  |  | Ah |
|  | 64 Onagraceae (1:1) |  |  |  |
|  | *Ludwigia prostrata* |  |  | Ah |
|  | 65 Polygonaceae (2:3) | 65 Polygonaceae (2:2) | 65 Polygonaceae (2:2) |  |
|  | *Polygonum muricatum* |  |  | Ah |
|  | *Polygonum persicaria* |  |  | Ah |
|  |  | *Polygonum hydropiper* | + | Ah |
|  | *Rumex acetosa* | + | + | Ph |
|  | 66 Umbelliferae (1:1) | 66 Umbelliferae (1:1) | 66 Umbelliferae (1:1) |  |
|  | *Daucus carota* | + | + | Ah |
|  | 67 Violaceae (1:1) |  |  |  |
|  | *Viola diffusa* |  |  | Ah |
|  |  | 68 Ranunculaceae (1:1) |  |  |
|  |  | *Semiaquilegia adoxoides* |  | Ah |
|  |  | 69 Tiliaceae (1:1) | 69 Tiliaceae (1:1) |  |
|  |  | *Grewia biloba* |  | S |
|  |  |  | *Corchoropsis tomentosa* | Ah |
|  |  | 70 Adiantaceae (1:1) | 70 Adiantaceae (1:1) |  |
|  |  | *Adiantum capillus-veneris* | + | Ph |
|  |  | 71 Begoniaceae (1:1) |  |  |
|  |  | *Begonia fimbristipula* |  | Ph |
|  |  | 72 Ephedraceae (1:1) |  |  |
|  |  | *Ephedra equisetina* |  | S |
|  |  |  | 73 Chenopodiaceae (1:1) |  |
|  |  |  | *Chenopodium glaucum* | Ah |
|  |  |  | 74 Hymenophyllaceae (1:1) |  |
|  |  |  | *Gonocormus minutus* | Ph |
|  |  |  | 75 Juncaceae (1:1) |  |
|  |  |  | *Juncus setchuensis* | Ph |

Ah: Annual herb; Ph: perennial herb; S: Shrub; T: Tree.

+ Indicated appeared species in any survey year (2008-2015).

^a^ The genera number within each family.

^b^ The species number within each family.
